# Supplementary material for: Exploring Hikikomori-like idiom of distress a year into the SARS-CoV-2 pandemic in Oman: Factorial validity of the 25-item Hikikomori Questionnaire, prevalence and associated factors
Source: PLoS One. 2023 Aug 7;18(8):e0279612. doi: 10.1371/journal.pone.0279612 (PMC10406187; doi:10.1371/journal.pone.0279612)
Supplement: Attachment — Submitted filename: Response to Reviewers.docx [file pone.0279612.s001.docx]

|  | Comments by the reviewers | Authors’ responses |
| --- | --- | --- |
| Reviewer #1 | Overall, the article is interesting, and it provides an insight on the development of the Omani version of the HQ-25 with the appropriate methodology. To strengthen the article, only a few issues need to be addressed. | Thank you for these comments. |
|  | •There are likely to have various Hikikomori-like idioms of distress (HLID) assessment tools available to the public, but the HQ-25 was purposefully chosen. The introductory section should provide the supporting details on this matter. | This important issue has now been narrated in the text. Please refer to the section highlighted in yellow in the *Introduction* section. |
|  | • In the sampling method section, the procedure to collect the data online should be more thoroughly explained | Thank you. Done as suggested. Please refer to the section highlighted in yellow in the *Materials and Methods* section, under subheading *2.4 Sampling Method*. |
|  | • The discussion section seems to well describe the findings, reflecting the practical ability of the Omani-version of the HQ-25 to detect prevalence and risk factors that were able to predict HLID in the Oman sample. | Thank you for these comments |
| Reviewer #2: | 1-due to use odd ratio, thus the authors should consider about sampling size. | This manuscript included 3 tasks: 1) Explore the prevalence rate of HLID in the general population in Oman, 2) Validate the HQ-25 in a local context, and 3) Determine risk factors associated with HLID. We agreed that we should consider different sample sizes to address different tasks. However, we included all three results in this manuscript. So, we chose the primary one - the prevalence rate - because this is the first population-based study conducted in Oman. This could potentially be a limitation, so we narrated this in the limitations section and suggested that we should consider different samples on both validity of HQ-25 and the risk factor (odds ratio) with HLID, highlighted in yellow in the *Limitations* subsection of the *Discussion*. |
|  | 2-According first commnet, the auhors should examine the assumption of using an odd ratio. | We agreed that using OR is suitable for a case-control study instead of a cross-sectional one. As we stated, this is the first population-based study conducted in Oman, and all associated risk factors with HLID in this report may be misinterpreted. We narrated this as one limitation (highlighted in yellow) and suggested that a case-control study would be highly recommended for further studies. |
|  | 3-well revise and well organized article. | Thank you for these comments |
