# Supplementary material for: Exploring Hikikomori-like idiom of distress a year into the SARS-CoV-2 pandemic in Oman: Factorial validity of the 25-item Hikikomori Questionnaire, prevalence and associated factors
Source: PLoS One. 2023 Aug 7;18(8):e0279612. doi: 10.1371/journal.pone.0279612 (PMC10406187; doi:10.1371/journal.pone.0279612)
Supplement: Attachment — Submitted filename: point-counterpoint.docx [file pone.0279612.s002.docx]

| Reviewer |  | Authors’ respond |
| --- | --- | --- |
| Reviewer #1: | The article appears to have been revised in response to earlier suggestions; thus, the manuscript is suitable and acceptable for publication in the journal. | Thank you for your review and positive feedback on the revised version of our manuscript. We appreciate the time and effort you have taken to provide us with your valuable suggestions, which have helped us to improve the quality of our work |
| Reviewer #2: | The authors should explain the method for EFA rotation and should show the R-sqaure statistic in the result. very well revise | Thank you for your comments. We follow the rotation promax method as suggested by Teo et al. 2018. We used this rotation method to ensure that we could directly compare with the results from Teo et al., 2018. It was included in the 2.7. Statistical analysis section. For the R-square(=0.20), we included it in the results section highlighted in yellow.. |
| Reviewer #3: | Thank you for allowing me to contribute to the review of this manuscript. I believe this study has the strength of providing some data on the characteristics of hikikomori in Oman but also has the weakness of being unclear about the definition of hikikomori and the appropriate interpretation of the analysis results. I hope these comments will help. | Thank you for taking the time to review our manuscript and for your valuable comments. We appreciate your feedback on the strengths and weaknesses of our study, and we are pleased to hear that you found the data on the characteristics of hikikomori in Oman to be a strength. We have responded to the esteemed reviewer’s comments as detailed below. We appreciate your helpful suggestions, and we are confident that our revisions will improve the quality of our work. |
|  | Comment # 1. The definition of hikikomori in this study is crucial. Apart from the cutoffs of the questionnaire, hikikomori in this study should be defined. As the authors state in the Limitation, the cutoff for the HQ-25 Omani version has yet to be validated. In the Introduction, I suggest that the authors clarify the definition of hikikomori in this study and explain why they have chosen it. | Thank you for your valuable feedback. We agree that the definition of hikikomori is crucial. The text has been narrated to address this particular suggestion. |
|  | Comment # 2. Hikikomori is naturally associated with occupational status. Therefore, when comparing hikikomori between emerging and developed economies, the authors should also focus on differences in socioeconomic factors in those countries. I suggest the authors review previous studies and add how they recognize the association between socioeconomic factors and hikikomori. | Thank you for your comment. We appreciate it. We would like to note that the main focus of our study was to investigate the factorial validity of the 25-item Hikikomori Questionnaire, as well as the prevalence and associated factors of hikikomori. While we did explore socio-economic status, it was only found to be significant in the univariate associations and not in the logistic model. |
|  | Comment # 3. This study distributed the questionnaire across multiple social media platforms. Many participants may have used more than one platform. Did the authors have procedures to prevent one participant from responding more than once? | Thank you for raising this question. The measures we have taken to prevent one participant from responding more than once have been in the text. |
|  | Comment # 4. The authors might want to declare no multicollinearity in logistic regression analysis. | We added this statement in the results section and now highlighted in the document. |
|  | Comment # 5. In Table 2, the data from Teo et al. (2018) are probably the "Japanese" version, not the HQ-25 "English" version. | Thank you. Used HQ-25 that was published in English as reported here (<https://www.dralanteo.com/services-2>) as this article (Teo AR, Chen JI, Kubo H, Katsuki R, Sato-Kasai M, Shimokawa N, et al. Development and validation of the 25-item Hikikomori Questionnaire (HQ-25). Psychiatry and Clinical Neurosciences. 2018;72: 780-788. doi:https://doi.org/10.1111/pcn.12691.). |
|  | Comment #6:  6. 3.3. Confirmatory factor analysis The extremely high covariance (0.98) between Socialization and Isolation suggests that a two-factor or one-factor structure would be appropriate. I believe the authors should discuss whether this too-strong correlation, also reported by Amendola et al. (2022), is a problem with the HQ-25 or is specific to the Omani version. The authors might also want to provide fit index data for two-factor or one-factor models so that readers can compare them. Amendola, S., Presaghi, F., Teo, A. R., & Cerutti, R. (2022). Psychometric properties of the Italian version of the 25-item Hikikomori Questionnaire. International journal of environmental research and public health, 19(20), 13552. | Thank you for the comments. After splitting the data into half, the results of covariance were drop. See the new results on CFA. We agreed that the current Arabic version need to have more iin-depth revision especially the cultural issues. The team will continue it to improve the content of this version. |
|  | Comment #7 7. I agree with the authors that the lack of validity of the scale is a limitation of this study. Therefore, the risk factors results may also need to be interpreted cautiously. I think the authors need to emphasize this in Limitation. Without understanding the validity of this scale as a screening tool for hikikomori, it may incorrectly identify risk factors or fail to recognize them. The high rate of HLID in this study compared to previous studies also supports this concern. | Thank you for your suggestion. We have incorporated the concerns raised by the esteemed reviewers into the revised text. |
|  | Comment # 8. The authors hypothesized that the COVID-19 pandemic would influence the HLID epidemic. Therefore, the authors might want to add the influence of this pandemic on the results of the EFA and CFA. If telework and online learning have become more prevalent and people spend more time at home, would the correlation between the Isolation factor and other factors have been lower than before the pandemic? | This suggestion to investigate the influence of the pandemic on the correlation between the Isolation factor and other factors is insightful. The prevalence of telework and online learning, along with increased time spent at home during the pandemic, could potentially impact the strength of correlations. However, it may be premature at this stage to fully examine the influence of the COVID-19 pandemic on the results of the exploratory factor analysis (EFA) and confirmatory factor analysis (CFA). Future research efforts should aim to investigate the influence of the pandemic on the HLID epidemic and further explore the correlations between the Isolation factor and other factors in the specific context of increased telework and online learning. |
|  | Comment # 9. Lines 534-535 When comparing the characteristics of hikikomori with other studies, the definition of hikikomori should also be compared. For example, in Yong et al. (2020), 47% of the "hikikomori" in this study are full-time workers, although many are certainly married. Since hikikomori are often regarded as those not working (Nonaka et al., 2022), the definition by Yong et al. (2020) differs between these and hikikomori. The high proportion of married people may also just reflect the difference in definitions. Nonaka, S., Takeda, T., & Sakai, M. (2022). Who are hikikomori? Demographic and clinical features of hikikomori (prolonged social withdrawal): A systematic review. Australian & New Zealand Journal of Psychiatry, 56(12), 1542-1554. | Thank you for your valuable feedback. We agree with the reviewer's suggestion that it is important to compare the definition of hikikomori used in our study with those used in other studies. |
|  | Comment # 10. There appears to be duplication on lines 185 and 200. | Thank you. Indeed there are duplications. The text has been reconciled. |
|  | Comment # 11. There appear to be some inappropriate citations. An overall check is needed. For example, the following is written in line 503: "For example, in a study in China by Yong et al. [88], it was reported that men are more prone to having severe symptoms of HLID compared to females." However, Yong et al. is a study conducted in Japan. Furthermore, it does not match the list of references. “89. Yong RK, Fujita K, Chau PY, Sasaki H. Characteristics of and gender difference factors of hikikomori among the working-age population: A cross-sectional population study in rural Japan. Nihon Koshu Eisei Zasshi. 2020;67(4):237-246.” | Thank you for uncovering the typos. With a recommendation from reviewer 6 [ see Comment #6(“It is not clear why the authors spend an entire paragraph discussing the gender difference, which was only significant in the univariate associations and not in the joint model”)]. We agree with the esteemed. To reduce word count, this particular discussion was removed. The cut-down here is consistent with Reviewer 3 (Comment # 8: “The Discussion is very long”). |
|  |  |  |
| Reviewer #4: | Comment # 1: The paper by Al-Sibani et al taps a novel and relevant issue, dealing with the detection of hikikomori(-like idiom of distress, HLID) in general population (of Oman, in the specific case). Authors report on the results of a snow-ball survey, based on the newly translated Arab version of HQ-25, in which 44% of the 1529 subjects expressed HDLI. They conclude on the good validity of the scale and on the possible role of several social and demographic factors may explain the presence of HLID. | Thank you for taking the time to review the revised version of our manuscript and for providing us with your valuable feedback. We greatly appreciate your efforts and suggestions which we have amended as much as possible. Please see below. |
|  | Comment # 2: The paper is written in a good English and its structure is clear. | Thank you for your support |
|  | Comment # 3: The authors should better explain the recruitment procedure (they report to have recruited 1529 subject, with a high response rate [more than 75%] in the very short period of two weeks). To exclude the risk of sampling bias, Authors should report in more detail within the manuscript the channel they used to publicize the study and all related recruitment procedures. | Thank you for your valuable feedback on our manuscript. We appreciate your suggestion to report in more detail within the manuscript about the channels and recruitment procedures we used to publicize the study. Some of the measures to avoid duplication have already been asked by another reviewer and we have already responded as detailed above (Reviewer 3, Comment #3). Another reason for accruing a reasonable response rate is that this study is focus highly focused study with a very specific outcome measure and it was done in a society with high internet penetration with high utilisation of the WhatsApp application (https://timesofoman.com/article/98544-whatsapp-is-the-most-popular-social-media-platform-in-oman#:~:text=Muscat%3A%20WhatsApp%20is%20the%20most,them%20using%20WhatsApp%20in%202020.). One unique feature that might have enhanced participation is the use of social medial influence. This has been now highlighted in the text. Further, this study has employed snowball techniques. it was therefore equipped to encourage recipients to cascade the link to others in their network can also increase the reach of the survey. However, there are likely to be two sources of attrition. These two sources of attrition have been narrated in the text as part of the limitation. Otherwise, in various parts of the text, we have explained the other factors that could contribute to recruitment. While online surveys are increasingly being used to gather data on community trends, it remains to be seen whether snowball techniques are more effective at accruing a higher response rate or if there are sociocultural factors that influence response rates. Therefore, further empirical investigation of online recruitment methods is warranted. |
|  |  |  |
| Reviewer #5: | #1.Thank you for the opportunity to review this study that attempts to examine the factor structure of an Arabic language version of the HQ-25 in an internet derived sample from Oman. There is a lot of good material in this paper. Unfortunately, it has some significant flaws and attempts to achieve more than is justified with the available data.  I would encourage the authors to consider re-writing the work as a more focused and psychometrically rigorous cross-cultural scale development study.  The following comments and observations are offered in the hope that they may help improve future versions of the paper. | Thank you very much for taking the time to review our study and for your positive feedback on our work. We appreciate your acknowledgement of the efforts we put into examining the factor structure of the Arabic version of the HQ-25 in an internet-derived sample from Oman. We will attempt to address the flaws that are highlighted below. The attempts to achieve more than is justified with the available data’ emerged from the myriad of reviewers who critique the conceptual issues which resulted in achieve more than is justified with the available data. We are happy that your critiques are legitimate and direct deal with the factor structure of an Arabic language version of the HQ-25 which essence of this manuscript |
|  | Comment # 2: There are some odd analytic choices, most notably the decision to conduct a CFA on exactly the same sample that was used to do the initial EFA.. | Thank you for this comment. We agreed that the data for EFA and CFA should be separated. Please refer to the new results on EFA and CFA. |
| 1. Introduction | Comment # 3: The introduction presents a comprehensive and clear account of how factors such as rapid modernisation and economic development combined with society level shocks like the COVID19 pandemic may provoke arise in behavioural phenotypes such as extreme social withdrawal. There is also a good argument made for the choice of the HQ-25 as the measure that is the focus of this study. In many ways, this is the strongest element of the paper. | Thank you very much for taking the time to review our study and for your positive feedback on the introduction section of our paper. We are pleased to hear that you found the account of how societal factors, such as rapid modernization and economic development, may contribute to behavioural phenotypes such as extreme social withdrawal, to be clear and comprehensive. |
|  | Comment # 4:  Materials and Methods Line 206: It seems that the initial sample was obtained via recruitment through social media platforms followed by snowball recruitment. Please provide more information about how the initial point of recruitment worked and any evidence that this ensured an epidemiologically representative sample. | Thank you for bringing up this issue, which has also been raised by other reviewers. We have carefully considered the comments provided by Reviewer 3 (Comment #3) and Reviewer 4 (Comment #3), and have addressed the concerns related to our recruitment methods. We recognize the importance of these issues and have acknowledged them as limitations of our study. Once again, thank you for your feedback, which has helped us to improve the quality of our work. |
|  | Comment # 5:  Line 232: The description of the HQ-25 needs careful editing (also noted in the “Typo’s section below). For example, the coefficients for the psychometric properties of the original scale should be presented so that they can be compared to the patterns seen in the current cross-cultural validation of the instrument. | Thank you. These issues have now been addressed in the text as detailed below. |
|  | Comment # 6:  3. Results Lines 310-312 and Table 1: It is not clear if the information about mental illness reflects a current diagnosis or any lifetime experience of mental illness (that may now be remitted). Please clarify. | The information provided in the manuscript refers to both current and life-time experiences of mental illness. To address this concern, we have revised the manuscript in the method. |
|  | Comment # 6:  Lines 317-366: The authors approach to examining the scale factors was to conduct both an EFA and CFA on the same sample. This violates the standards of good practice for CFA and it is not clear why the authors did not split the sample and perform the EFA first and then attempt CFA on the second half. A clear and rigourous defence of the analytic approach taken is needed before this will be at publishable standard. | Thank you for your comments. We split the data in half to run CFA and EFA separately. Results are similar by using the whole data set. |
|  | Comment # 7 (A):  Line 386: The column header for Table 4 which states "Yes or No" needs to be edited to make it clear that this refers to falling above or below the cut off for significant Hikikomori features using the HQ-25 cut offs. | Thank you. This point has been addressed. The case-ness to HLD or otherwise has now been detailed. The cut-off is also explained via an asterisk with the confinement of the Table |
|  | Comment # 7 (B):  There should also be a more robust defence of using these cut-offs given that they were derived from scores obtained with non-Omani respondents. The use of the 42/100 cut-off point is mentioned in the discussion but it needs to be defended as part of the methods | Thank you. We have added more statements on the sensitivity, specificity and positive predictive value of HQ-25 as defined by the progenitor of this instrument (doi:https://doi.org/10.1111/pcn.12691). On one hand, the factorial analysis appears to be adequate. On the other, let’s not overlook the contentious issue is not cut-off point but rather what could be a spurious rate of HLID. We agree though both issues will require further research. |
|  | Comment # 8:  4. Discussion  The Discussion is very long and provides a further in-depth narrative that explores why Hikikomori-like Idioms of Distress in Oman may be best seen as a culture reactive phenomenon that is attributable to rapid modernisation, social change, and the impact of the pandemic. Unfortunately, the links back to the data are not as close as they should be and in the analysis of the limitations the authors acknowledge that because they did not measure duration of withdrawal behaviours there is considerable uncertainty about the epidemiological meaning of the presented data. Taking a more focused approach that concentrated on best practice measurement development would have produced a much more defensible paper that could have provided a springboard for next phase studies in Arabic speaking samples (e.g. determining the most sensitive cut-off point on the scale and confirming the factor structure in a second independent sample). | The comment suggests that the reviewer would have preferred a more focused approach, concentrating on best practice measurement development. However, we believe that our revised version now incorporates best practice measurement development. We have also addressed the statistical approach as suggested by the reviewers and acknowledged the limitations in the discussion. These limitations could provide a foundation for the next steps, including determining the most sensitive cut-off point on the scale and confirming the factor structure in a second independent sample. |
|  | Comment #9  5. Typographical Errors and Areas for Clarification  Lines 195 and 196 – “…this study required at least 1695 to 1958 samples…”. Please replace “samples” with “respondents” (this pattern persists across the entire manuscript – please revise all instances). | Valid point- Thank you. The term sample/s has been replaced by respondents in the text throughout the manuscript. |
|  | Comment #10  Lines 235-236 – Please amend the phrase “HQ-25 is a self-reported instrument of Likert types with a scale ranging from 0…” to reflect that the response options were presented in Likert format. | Thank you. Done as suggested |
|  | Comment #11  Line 237 – “The progenitor of this scale ranges from 0-100” – “progenitor” does not make sense in this context – please edit for clarity. | Thank you. Correction is done in the text |
|  |  |  |
| Reviewer #6: | Comment #1  The authors present results of a study in Oman that explores the structure of the Hikikomori Questionnaire (HQ-25) translated to Arabic, then confirms the structure and assesses potential risk factors for Hikikomori-like idiom of distress (HLID). Data were acquired through an on-line questionnaire 1 year after the Covid-19 shutdown in Oman. The manuscript will be strengthened if the authors consider the following points. | Thank you very much for taking the time to review our study and for your feedback on the manuscript. As detailed below, have considered the points you have raised and amended our manuscript accordingly. |
|  | Comment # 2  1. Authors report risk ratios in the abstract, tables and text of the manuscript. However, they have conducted a logistic regression, which provides estimates of odds ratios, not risk ratios. Authors should re-interpret their results in terms of odds rather than probability (or likelihood) and identify the results as odds ratios. | Thank you for this comment. We agreed that, we should change it to OR instead of risk factor. So we did |
|  | Comment # 3  2. It is unclear why authors use a variety of tests/approaches for the univariate analysis when the ultimate goal is to develop a joint logistic regression model - why not just fit simple logistic models with a single predictor and consistently report the odds ratio for all variables? That makes it easier to compare with the results from the multiple logistic regression model. | Thank you for your comment. We used a logistic model for univariate and multivariate regression. |
|  | Comment # 4 (A)  3. Authors need to be careful in their interpretation of numbers in tables, specifically when noting the subgroup the percentage is referring to. For example, line 310, authors state that of those with HLID, a certain percentage have various mental illness. However, it appears as though the sentence is referring to Table 1 which is for the entire sample. | Thank you for these comments. We have re-examined line 310 and it appears the presentation has typos. They were corrected. |
|  | Comment # 4 (B)  In line 512, authors say of 1035 female participants 71% (n=478) had HLID - 478/1035 is 46.2% (the 71% comes from Table 4, which reports column percentages...so of those with HLID, 71% were female. The same mistake is made in lines 565-566 | This point has been addressed as per your suggestion (Comment #6 “5. It is not clear why the authors spend an entire paragraph discussing the gender difference, which was only significant in the univariate associations and not in the joint model). So we delete the paragraph. Thank you |
|  | Comment #5  4. Table 4: the univariate results do not seem to match the numbers that are presented in the Yes and No columns. For example, for marital status, the result in the table is 6.64, but with the frequencies presented, the OR would be 2.81, while the risk ratio would be 1.73. Some of the reported numbers are actually odds ratios (though they are referred to as risk ratios, which as mentioned in point 1 above, are not the same), while others, I cannot tell how they are calculated, so likely are not either risk ratios or odds ratios. | Thank you for your comments. All updated and we used OR not RR. |
|  | Comment #6  5. It is not clear why the authors spend an entire paragraph discussing the gender difference, which was only significant in the univariate associations and not in the joint model. | We agree with this recommendation. The concerned section of the discussion has been removed. This is consistent with other reviewers who suggested that the discussion might be too long. Thank you for raising this point |
|  | Comment #7  Minor points: 1. line 43: "third is to prevalence" is awkwardly phrased | Thank you. Done |
|  | Comment # 8:  2. lines 147-148: "Nationwide, ..." is an incomplete sentence | Thank you. Done |
|  | Comment #9:  3. line 176: replace "frequency" with "prevalence" | Thank you. Done |
|  | Comment # 10:  4. lines 201-203 is an incomplete sentence | The description of exclusion/inclusion criteria has been improved. Thank you |
|  | Comment # 11:  5. lines 238-239 - authors state that the cut-off of 42 is widely used to determine HLID, but they do not specifically state in the methods that they use this cut-off for the study. I didn't notice specific mention for their study until the discussion. | The method employed by Teo et al. [45] sensitivity and specificity using receiver–  operator curve (ROC). This has been now narrated in the text. |
|  | Comment #12  6. line 271: EPA should be EFA | Thank you. Done |
|  | Comment #13 7. lines 290-291 - the good model fit values for CFI are stated in lines 289-290, so do not need to be restated. | Already removed |
|  | Comment # 14  8. line 372: "present of HLID" should be "presence of HLID" | Thank you. Done |
|  | Comment #15  9. lines 400-401: this is an incomplete sentence | We agree and the sentences has been amended. |
|  | Comment #16  10: line 446: "gold-stand" should be "gold-standard" | Thank you. Done |
|  | Comment #17  11. line 618: "less less inflated" should just be "less inflated" | Thank you. Done |
|  | Comment #18  12: line 637: "prevalence rate of HLID" should just be "prevalence of HLID" | Thank you. Done |
|  |  |  |
