# Supplementary material for: Exploring Hikikomori-like idiom of distress a year into the SARS-CoV-2 pandemic in Oman: Factorial validity of the 25-item Hikikomori Questionnaire, prevalence and associated factors
Source: PLoS One. 2023 Aug 7;18(8):e0279612. doi: 10.1371/journal.pone.0279612 (PMC10406187; doi:10.1371/journal.pone.0279612)
Supplement: Attachment — Submitted filename: point counterpoint.docx [file pone.0279612.s003.docx]

|  | Reviewer’s comment | Authors’ responses |
| --- | --- | --- |
| Reviewer #2 | 1-the authors should describe the method or technique that was used for setting sampling size in topic 2.4 | Thank you for your comments. We described how we came up with the sample size, including the expected outcome, precision, 95% CI, and the software we used. Please refer to 2.2 Power analysis. |
|  | 2-The snowball technique that is not suitable for CFA and inferential statistic that were used in this study. | Thank you for your comments. We agreed that the snowball technique is a non-probability sampling that might induce sampling bias. This will affect the factor/construct of the HQ-25 in CFA/inferential statistics, and we added this in the limitation highlighted in yellow. |
|  | 3-The reseult of this study (in case of CFA) should show or describe the model modification. | Thank you for this suggestion. However, we did not conduct any model modification by removing or adding more factors. We know that (from question 2) the sampling method will affect the factor in CFA/EFA, so the team decided not to do further analysis. In addition, we want to compare the HQ-25 results between Oman and Teo’s study (Teo et al., 2018), so we used the 3-factors. |
| Reviewer #3 | I believe the authors have adequately addressed many of my previous comments.  However, the following still needed to be adequately addressed. | We appreciate your acknowledgement. As detailed, we have carefully reviewed your additional comments and have made further revisions to address your remaining concerns. Thank you |
|  | 1. For multicollinearity, a statistic such as VIF should be provided. | As suggested, we included the VIP for each variable in the multivariate logistic model. The cut-off VIF > 5 indicates problematic (Menard S. Applied logistics regression analysis. 2nd Edn., SAGE Publications, Inc.). We added in Table 4, 2.7 Statistical analysis, and references were highlighted in yellow. |
|  | 2. In the Methods and Discussion of the HQ-25 article by Teo et al. (2018), it is clearly stated that they used the Japanese version (e.g., "Of note, Japanese was used for the administration of the scale." (p. 785)). The authors should carefully check that. | We agree. The text has been revised. Thank you. |
