# Supplementary material for: Exploring Hikikomori-like idiom of distress a year into the SARS-CoV-2 pandemic in Oman: Factorial validity of the 25-item Hikikomori Questionnaire, prevalence and associated factors
Source: PLoS One. 2023 Aug 7;18(8):e0279612. doi: 10.1371/journal.pone.0279612 (PMC10406187; doi:10.1371/journal.pone.0279612)
Supplement: Attachment — Submitted filename: Response to Reviewers.docx [file pone.0279612.s004.docx]

1. Please include a figure label and title for Figure 1 in your main manuscript

These are shown in lines 361-362 (page 14)

2. Your ethics statement should only appear in the Methods section of your manuscript. If your ethics statement is written in any section besides the Methods, please delete it from any other section.

Thank you. Done as suggested
